# Prolactin‐adjusted inferior petrosal sinus sampling: Pituitary and ectopic adrenocorticotropic hormone‐dependent Cushing syndrome

**DOI:** 10.1111/jne.70066

**Published:** 2025-08-05

**Authors:** Vera E. Sprenkeler, Antonius E. van Herwaarden, Annenienke C. van de Ven, Benno Kusters, Sjoerd F. M. Jenniskens, Romana T. Netea‐Maier, Joanne M. de Laat

**Affiliations:** ^1^ Department of Internal Medicine, Division of Endocrinology Radboud University Medical Center Nijmegen The Netherlands; ^2^ Department of Laboratory Medicine Radboud University Medical Center Nijmegen The Netherlands; ^3^ Department of Pathology Radboud University Medical Center Nijmegen The Netherlands; ^4^ Department of Medical Imaging Radboud University Medical Center Nijmegen The Netherlands

**Keywords:** ACTH hypersecretion, petrosal sinus sampling, prolactin adjustment

## Abstract

Inferior petrosal sinus sampling (IPSS) is a diagnostic procedure used to differentiate between ectopic adrenocorticotropic hormone (ACTH)‐dependent Cushing syndrome (EAS) and pituitary ACTH‐dependent Cushing syndrome (CD). This study investigated the diagnostic value of IPSS, focusing on the use of prolactin adjustments and different calculation methods. We retrospectively analyzed data from patients with ACTH‐dependent Cushing syndrome and inconclusive pituitary‐MRI who underwent IPSS with corticotropin‐releasing hormone (CRH) stimulation between 2015 and 2025. The cohort included 19 patients (16 CD, 3 EAS), with diagnoses confirmed by pathology examination and/or biochemical remission 1 year post‐surgery. A pituitary source was confirmed in all patients with CD (*n* = 16) through pathology and/or biochemical remission. An ectopic source was confirmed by pathology in two of three patients with EAS. Using unadjusted ACTH ratios and previously established cut‐off values resulted in three incorrect diagnoses out of 20 procedures. In contrast, prolactin‐adjusted peak ACTH ratios provided a more distinct separation between CD and EAS, enabling correct diagnosis in all cases. Optimal cut‐off values determined by receiver operating characteristic curve analysis were 1.0 for basal and 1.7 for concurrent prolactin‐adjusted peak ACTH ratios, yielding 100% sensitivity and specificity. Basal prolactin‐adjusted peak ACTH ratios were >1.5 in all patients with CD and <0.6 in all patients with EAS, while concurrent ratios were >1.1 in all patients with CD and <0.3 in all patients with EAS. Prolactin‐adjusted peak ACTH ratios improve the diagnostic accuracy of IPSS and can effectively differentiate between ectopic and pituitary sources of ACTH. This study enhances the diagnostic accuracy of inferior petrosal sinus sampling (IPSS) for differentiating pituitary from ectopic ACTH‐dependent Cushing syndrome by incorporating prolactin measurements and exploring various calculation methods. The findings contribute to advancing diagnostic techniques and improving clinical management of endocrine disorders. By enabling more accurate identification of the underlying cause of ACTH‐dependent Cushing syndrome, this work supports clinicians in selecting optimal treatment strategies.

## 1 INTRODUCTION

Cushing syndrome comprises a large set of symptoms that reflect prolonged and inappropriately high exposure to glucocorticoids. Non‐iatrogenic Cushing syndrome is uncommon, with an estimated incidence of 0.2–5 million per year. The overproduction of cortisol may be due to an excess of adrenocorticotropic hormone (ACTH) secretion or by autonomous adrenal overproduction.^1^ ACTH‐dependent hypercortisolism is typically due to a pituitary source (80%–85%) or, less commonly, an ectopic source (10%–20%).^2^, ^3^ Differentiating between pituitary and ectopic ACTH secretion is crucial for determining appropriate treatment, such as transsphenoidal resection of a pituitary tumor or resection of an ectopic tumor. This distinction is particularly challenging when a pituitary microadenoma is not clearly visible on MRI.^4^

Inferior petrosal sinus sampling (IPSS) is used to distinguish pituitary ACTH‐dependent Cushing disease (CD) from ectopic ACTH‐dependent syndrome (EAS) after ACTH‐dependent hypercortisolism is confirmed biochemically and no adenoma or an adenoma <6 mm is detected on MRI.^4^, ^5^, ^6^, ^7^ According to updated guidelines by Fleseriu et al., management of adenomas 6–9 mm remains debated, with some recommending IPSS for adenomas <10 mm.^8^ An (unadjusted) petrosal sinus‐to‐peripheral ACTH ratio ≥2.0 at baseline or ≥3.0 post‐corticotropin‐releasing hormone (CRH) administration indicates a pituitary source, while lower ratios suggest an ectopic source.^8^, ^9^ However, these cut‐off values are based on limited studies with small cohorts,^2^, ^9^, ^10^, ^11^, ^12^, ^13^ and their generalizability is uncertain. Factors such as fluctuations in ACTH secretion, catheter placement relative to the ACTH source, and variable venous efflux can affect ACTH concentrations and calculated ratios.^2^, ^6^, ^9^, ^14^, ^15^ Consequently, IPSS should ideally be standardized and performed in experienced centers to minimize variability. Reported false‐negativity rates for IPSS range from 1% to 12%.^9^, ^16^, ^17^

To mitigate these limitations, prolactin measurements have been proposed to enhance IPSS outcomes. Recent studies suggest that prolactin‐adjusted ACTH ratios improve diagnostic accuracy,^2^, ^9^, ^10^, ^13^, ^14^, ^18^, ^19^ though different adjustment methods and cut‐off values have been used.^2^, ^9^, ^10^, ^15^

Prolactin may help normalize variability in catheter placement and venous efflux, potentially improving IPSS diagnostic accuracy. However, the performance of various prolactin adjustment methods in real‐life clinical practice remains unclear.^15^, ^20^ The aim of this study is to evaluate whether the use of prolactin ratios improves IPSS accuracy in clinical practice at a center of expertise for pituitary tumors, by comparing different prolactin adjustment methods.

## 2 METHODS

### 2.1 Participants

We retrospectively collected data of all patients with ACTH‐dependent Cushing syndrome who underwent IPSS at Radboud University Medical Center between 2015 and 2025 (*n* = 32). Patients lost to follow‐up (*n* = 3) and those who underwent stimulation with desmopressin (DDAVP) instead of CRH (*n* = 10) were excluded from the main analysis. One patient underwent IPSS twice, resulting in 20 IPSS procedures for the main analysis. Six DDAVP‐stimulated IPSS procedures were included in an additional analysis; four other DDAVP‐stimulated procedures were excluded due to follow‐up <1 year or pending surgery. Diagnosis of Cushing syndrome was based on clinical symptoms and biochemical tests, including lack of suppression of fasting morning cortisol (>0.05 μmol/L) after a 1 mg dexamethasone test, elevated 24‐h urinary free cortisol, and increased midnight salivary cortisol.^4^ All included patients had inconclusive pituitary‐MRI findings, showing no adenoma (*n* = 11) or adenomas <6 mm (*n* = 8). Ethical approval from our institutional research board (METC Oost‐Nederland: 2022‐13758) was obtained before the start and this study was conducted in accordance with the Helsinki Declaration. Informed consent was obtained per RadboudUMC guidelines for research using previously collected clinical data and materials.

### 2.2 MRI protocol

Pituitary‐MR imaging was performed on a 3‐Tesla clinical MR imaging system, Magnetom Skyra; Siemens Healthineers, Erlangen, Germany. Standardized pituitary‐MRI protocol consisted of T1‐tse‐sagital, T1‐se‐coronal‐bloodsuppression, and T2‐tse‐coronal images. Post‐contrast (Dotarem: 0.2 mL/kg, max. 15 mL, flow 2.5 mL/s): T1‐Vibe‐coronal‐dynamic, T1‐tse‐sagital, and T1‐se‐coronal‐bloodsuppression images. Slice thickness was 3.0 mm for T1‐tse‐sagital, 3.05 mm for T1‐tse‐sagital post contrast, and 2.0 mm for all other described images.

### 2.3 IPSS procedure

**FIGURE 1:**
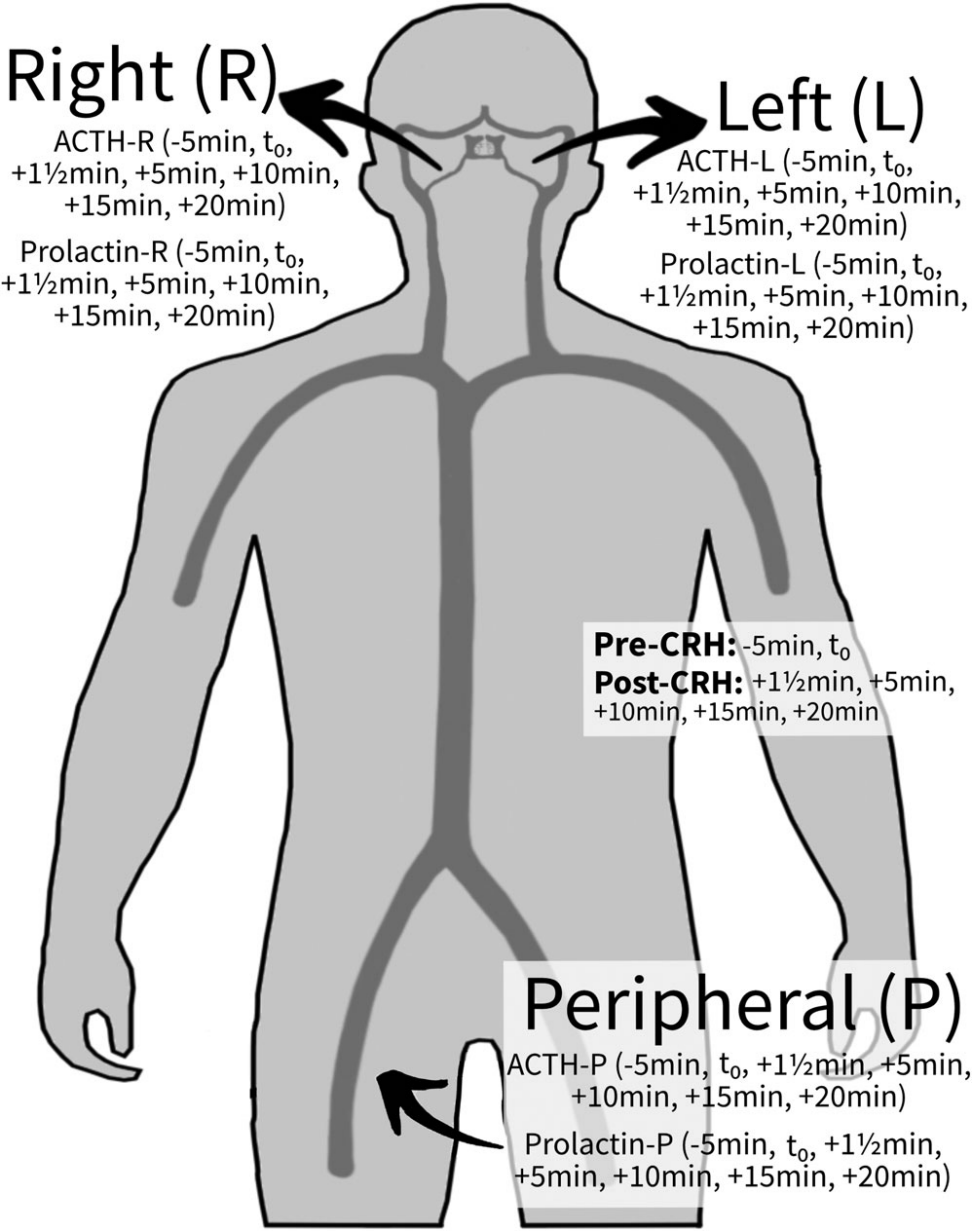
During IPSS, blood samples are obtained simultaneously from the left inferior petrosal sinus, right inferior petrosal sinus, and femoral vein at the following time points: −5 min, *t*_0_, +1½ min, +5 min, +10 min, +15 min, and +20 min.

Bilateral simultaneous catheterization of the inferior petrosal sinuses was performed, confirmed by venous angiography. Blood samples included a sample from both petrosal sinuses (referred to as the left and right ACTH values) and peripheral blood samples obtained from the left or right femoral vein. Samples were obtained 5 min prior (−5 min) and just before (*t*_0_) CRH was administered. After administering 100 μg CRH at *t*_0_, samples were collected at +1½, +5, +10, +15, and +20 min^21^ (Figure 1). ACTH values were measured immediately post‐procedure, and samples were stored at −30°C until prolactin was analyzed.

### 2.4 Hormone assays

ACTH and prolactin values were measured using an electrochemiluminescence immunoassay (ECLIA), measured on a COBAS E801 random‐access analyzer (Roche Diagnostics GmbH, Mannheim, Germany). The ACTH measuring range was 0.33–440 pmol/L, and the prolactin measuring range was 2–10,000 μIU/mL. The within‐laboratory coefficient of variation is 4.6% at 2.2 pmol/L and 2.5% at 22 pmol/L for ACTH and 3.0% at 160 μIU/mL and 3.0% at 880 μIU/mL for prolactin.

### 2.5 Interpreting IPSS

ACTH ratios were calculated as the petrosal sinus ACTH value divided by the concurrent peripheral ACTH value (Figure 1: ACTH‐L/ACTH‐P and ACTH‐R/ACTH‐P). The highest ratio across all time points, pre‐ or post‐CRH, was the peak ACTH ratio. A ratio ≥2.0 pre‐CRH or ≥3.0 post‐CRH indicated CD.^21^

For the prolactin‐adjusted ACTH ratios, first the prolactin ratios for both the left and right sides were calculated by dividing the left and right prolactin values by the concurrent peripheral prolactin value (Figure 1: prolactin‐L/prolactin‐P and prolactin‐R/prolactin‐P).

Prolactin adjustment was performed using two previously reported methods^2^, ^9^:

Method 1. Basal prolactin‐adjusted peak ACTH ratio = (peak post‐CRH^a^ ACTH ratio)/(ipsilateral basal[*t*_0_] prolactin ratio)
Method 2. Concurrent prolactin‐adjusted peak ACTH ratio = (peak^b^ ACTH ratio)/(ipsilateral concurrent prolactin ratio)

- Highest achieved ratio among all post‐CRH time points during the procedure.
- Highest achieved ratio among all time points during the procedure.

Hypothetically, the response of ACTH and prolactin on CRH stimulation may vary across time points. To investigate whether including non‐peak ACTH ratio calculations could improve the diagnostic value compared with adjusting only the peak ACTH ratio (Methods 1 and 2), we included two other calculation methods (Methods 3 and 4). Prolactin adjustments were made according to the formulas below for each 14 ACTH ratios —corresponding to the seven left and seven right sampling time points — after which the highest prolactin‐adjusted ACTH ratio was used for diagnosis. A detailed overview of the calculation Methods 1–4 has been added as Data S1.

Method 3. Basal prolactin‐adjusted ACTH ratio at *specific time point and side* = ACTH ratio at *specific time point* ^*a*^ *and side* ^*b*^/ipsilateral basal prolactin ratio
Method 4. Concurrent prolactin‐adjusted ACTH ratio at *specific time point and side* = ACTH ratio at *specific time point* ^*a*^ *and side* ^*b*^/ipsilateral concurrent prolactin ratio

- ACTH values obtained at *t* − 5 min, *t*_0_, +1½ min, +5 min, +10 min, +15 min, or +20 min were used.
- ACTH values obtained from the left or right petrosal sinuses were used.

### 2.6 Confirmation of the diagnosis

CD was confirmed by pathology of adenomatous tissue or biochemical remission 1 year post‐surgery, defined as negative 1 mg dexamethasone suppression test (cortisol in serum the morning after dexamethasone <0.05 μmol/L), low late‐night salivary cortisol, and/or morning serum cortisol <0.06 μmol/L.^5^, ^8^ EAS was confirmed by pathology of a neuroendocrine tumor or biochemical remission, alongside pituitary‐MRI without a lesion.

### 2.7 Statistics

Continuous and categorical variables were described as mean ± standard deviation (SD) and percentages. Fisher's exact tests were used to check for confounding by variables. The following subgroups were analyzed: male versus female, ≤50 and >50 years of age, and blood sample collection date ≤30 and >30 months. The median age and the median blood sample collection date were used to obtain subgroups of equal size for both variables. A *p*‐value <.05 was considered statistically significant. Optimal cut‐off values were obtained from receiver operating characteristic (ROC) curve analysis.

### 2.8 Literature search

A literature search of the PubMed database was conducted to identify studies involving prolactin measurement adjustments in IPSS. The following search terms were used: Cushing syndrome, ACTH hypersecretion, hypercortisolism, prolactin, and inferior petrosal sinus sampling. Publications up to January 2025 were considered. Articles reporting both unadjusted ACTH ratios and prolactin‐adjusted ACTH ratios were included to enable the construction of a contingency table, for example, true positives (TP), false positives (FP), false negatives (FN), and true negatives (TN). No restrictions were made based on sample size.

## 3 RESULTS

### 3.1 Patients' characteristics

Of the 19 patients included, 16 had a pathology and/or remission‐confirmed CD (age 44 ± 16 (SD) years, 12 women). All 16 patients underwent pituitary surgery; 8 were confirmed by both pathology and remission, 3 were confirmed by only pathology, and 5 were confirmed by only remission. Three patients had EAS (age 65 ± 3 (SD) years, two women). Two EAS cases had histologically confirmed ACTH‐producing neuroendocrine lung tumors, and one had an unknown source (case 18). Patient characteristics are summarized in Table 1. Peripheral prolactin levels documented before IPSS were available for 17 out of 19 patients and excluded a state of hyperprolactinemia in all but one patient. Fisher's exact tests showed no confounding by variables. Blood samples required for IPSS were obtained in 17/20 (85%) procedures; two had only right‐sided results, and one had a missing right‐sided value.

**TABLE 1:** Patients' characteristics.

Patient	Age (y) at time of IPSS	Sex	Peak basal (*t*_0_) unadjusted ACTH ratio	Peak unadjusted ACTH ratio post‐CRH	Pituitary‐MRI result	Postoperative biochemical remission for at least 1 year	Confirmed by pathology examination	Diagnosis
1	36	F	4.9	7.3	No adenoma	Indeterminate	Yes	Pituitary
2	41	F	49.2	117.1	Adenoma <6 mm	Yes	Yes	Pituitary
3	63	F	8.4	33.4	Adenoma <6 mm	Yes	Yes	Pituitary
4	65	M	5.9	19.7	No adenoma	Yes	Yes	Pituitary
5	35	F	25.0	71.2	No adenoma	Yes	Yes	Pituitary
6	36	M	5.0	141.1	Adenoma <6 mm	Yes	Yes	Pituitary
7	58	F	18.2	282.2	No adenoma	Yes	No	Pituitary
8	60	F	14.8	577.5	Adenoma <6 mm	Yes	No	Pituitary
9	20	F	37.7	186.4	No adenoma	No	Yes	Pituitary
10	41	F	26.5	526.7	No adenoma	Yes	No	Pituitary
11	49	F	33.1	367.0	No adenoma	Yes	No	Pituitary
12	49	M	0.8	2.6	Adenoma <6 mm	Yes	No	Pituitary
13 (2× IPSS)	26 (1st IPSS) 28 (2nd IPSS)	F	1.1 (1st IPSS) 10.4 (2nd IPSS)	1.7 (1st IPSS) 21.6 (2nd IPSS)	Adenoma <6 mm	Yes	Yes	Pituitary
14	58	F	15.8	87.3	No adenoma	Yes	Yes	Pituitary
15	14	M	23.0	106.7	Adenoma <6 mm	No^a^	Yes	Pituitary
16	66	F	1.2	4.3	Adenoma <6 mm	Yes	Yes	Pituitary
17	66	F	2.2	10.2	No adenoma	NA^b^	Yes	Ectopic
18	60	F	1.3	2.4	No adenoma	NA^b^	NA^c^	Ectopic
19	67	M	1.3	1.8	No adenoma	Yes	Yes	Ectopic

^a^ Transsphenoidal hypophysectomy <1 year.

^b^ Not applicable, patient had a bilateral adrenalectomy.

^c^ Not applicable, no pathology examination done.

### 3.2 Cut‐off values

#### 3.2.1 ACTH ratios

**FIGURE 2:**
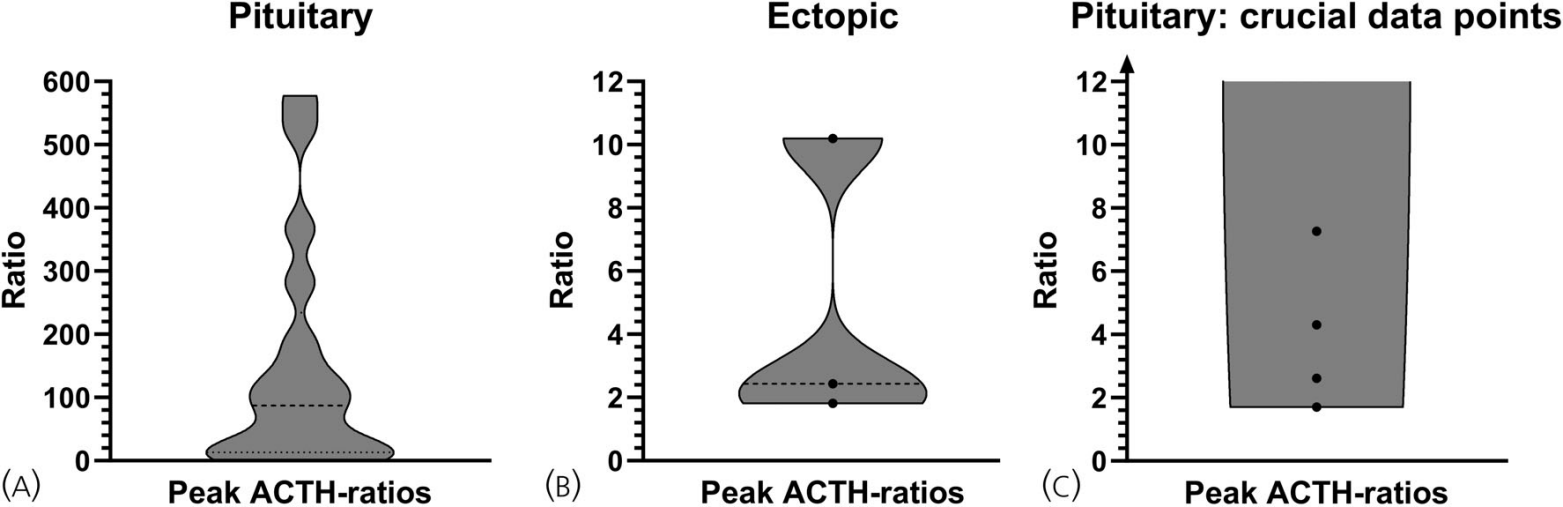
Distribution of the (unadjusted) ACTH ratios shown separately for the patients with pituitary ACTH‐dependent (A) and ectopic ACTH‐dependent Cushing syndrome (B). In (C) the lower section of (A) is shown, illustrating the crucial data points.

ACTH ratios were calculated for each time point, and peak ratios were compared between patients with CD and EAS. No clear cut‐off value diagnosed all patients. Peak ACTH ratios ranged from 1.7 to 577.5 for CD and 1.8 to 10.2 for EAS (Figure 2A–C). Using the standard cut‐offs (≥2.0 pre‐CRH; ≥3.0 post‐CRH),^8^, ^20^ sensitivity was 88.2% (2 false negatives: cases 12, 13), and specificity was 66.7% (1 false positive: case 17) (Data S2).

#### 3.2.2 Prolactin‐adjusted peak ACTH ratios

**FIGURE 3:**
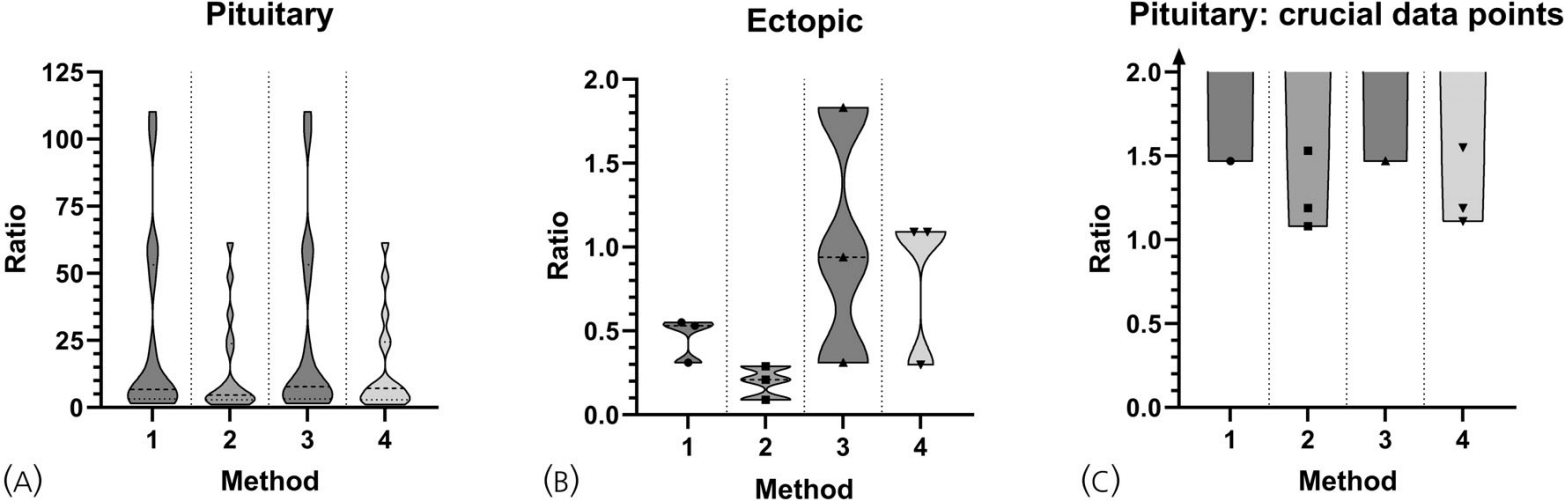
Distribution of the prolactin‐adjusted ACTH ratios calculated by different methods, shown separately for the patients with pituitary ACTH‐dependent (A) and ectopic ACTH‐dependent Cushing syndrome (B). In (C) the lower section of (A) is shown, illustrating the crucial data points; Method 1: Basal prolactin‐adjusted peak ACTH ratio. Method 2: Concurrent prolactin‐adjusted peak ACTH ratio. Method 3: Basal prolactin‐adjusted ACTH ratio. Method 4: Concurrent prolactin‐adjusted ACTH ratio.

**FIGURE 4:**
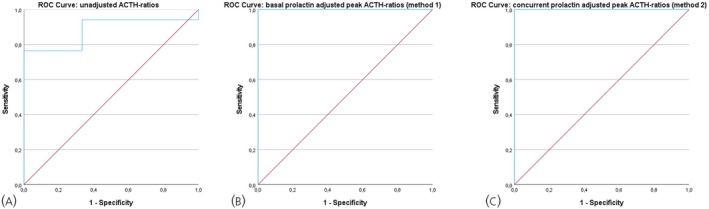
Receiver operating characteristic (ROC) curve for unadjusted ACTH ratios (A), basal prolactin‐adjusted peak ACTH ratios (B) and concurrent prolactin‐adjusted peak ACTH ratios (C).

Prolactin adjustment allowed clear discrimination between CD and EAS. Basal prolactin‐adjusted peak ACTH ratios (Method 1) were >1.5 in all patients with CD and <0.6 in all patients with EAS (Figure 3A–C). Concurrent prolactin‐adjusted peak ACTH ratios (Method 2) were >1.1 for CD and <0.3 for EAS. Optimal cut‐offs from ROC analysis were 1.0 (basal) and 0.7 (concurrent), both yielding 100% sensitivity and specificity (Figure 4A–C, Data S2). A consistent post‐CRH increase in prolactin on the ACTH‐dominant side (13.5–664.3%) was observed in patients with CD, influencing the adjusted ratios.

### 3.3 Preference for adjustment based on peak ACTH ratio

Adjusting all time‐point ACTH ratios and selecting the peak prolactin‐adjusted ratio (Methods 3 and 4) failed to discriminate CD from EAS (Figure 3A–C).

### 3.4 Timing of basal sample

No differences in IPSS interpretation were found when basal values at *t* − 5 min were used instead of *t*_0_ (Data S3; Table S1).

### 3.5 Discrepant results between unadjusted and adjusted ACTH ratios

#### 3.5.1 Case description patient 12

This patient was diagnosed with ACTH‐dependent hypercortisolism, and the pituitary‐MRI showed a right‐sided microadenoma of 5 mm. In 2016, this patient underwent IPSS, resulting in all ACTH ratios <2.0 pre‐CRH and <3.0 post‐CRH, consistent with an ectopic source. CT imaging of the thorax and abdomen showed no evidence of an ectopic source. Basal and concurrent prolactin‐adjusted peak ACTH ratios were, respectively, 2.8 and 1.2. The patient did not prefer to undergo an IPSS again. An explorative transsphenoidal hypophysectomy was performed, after which the patients' symptoms were reduced. Histological examination did not confirm a pituitary source, but biochemical remission after 1 year of follow‐up was established, and therefore, considered as confirmation of a pituitary origin. In addition, 8 years after surgery, the patient remains in remission.

#### 3.5.2 Case description patient 13

This patient was diagnosed with ACTH‐dependent hypercortisolism, and the pituitary‐MRI showed a hyperintense configuration of 5 mm in the midline of the pituitary, without a clear aspect of a microadenoma. In 2020, this patient underwent IPSS, resulting in all ACTH ratios <2.0 pre‐CRH and <3.0 post‐CRH (all between 0.9 and 1.7), consistent with an ectopic source. PET and CT imaging of the thorax and abdomen showed no ectopic source. Inhibition of cortisol production with metyrapone was initiated, and the patient was followed up. IPSS results were reassessed by adding prolactin adjustments, which revealed a basal and concurrent prolactin‐adjusted peak ACTH ratio of 1.5 and 1.1, respectively. After discontinuation of the medication, the IPSS was repeated in January 2022, which resulted in all right‐sided ACTH ratios ≥2.0 pre‐CRH and ≥3.0 post‐CRH (all between 7.5 and 21.6) and basal and concurrent prolactin‐adjusted peak ACTH ratios of 2.7 and 4.1, respectively, consistent with a pituitary source. In 2022, the pituitary‐MRI was repeated and showed a pituitary adenoma. A transsphenoidal hypophysectomy was performed, and histological examination confirmed a pituitary source. One year after surgery, the patient remains in remission, with morning cortisol of 0.07 μmol/L requiring hydrocortisone substitution.

#### 3.5.3 Case description patient 17

This patient was diagnosed with ACTH‐dependent hypercortisolism, and the pituitary‐MRI showed no adenoma. In 2020, this patient underwent IPSS, resulting in all right‐sided ACTH ratios ≥2.0 pre‐CRH and ≥3.0 post‐CRH, consistent with a pituitary source. Basal and concurrent prolactin‐adjusted peak ACTH ratios were, respectively, 0.5 and 0.1, consistent with an ectopic source (Data S4; Figure S1). The patient underwent a transsphenoidal hypophysectomy; however, no histological evidence was found for an ACTH‐producing pituitary adenoma. Hypercortisolemia persisted after the pituitary surgery, and therefore, a bilateral adrenalectomy was performed. Six months later, a neuroendocrine lung tumor was discovered. Histological examination showed a small number of ACTH‐producing cells in this neuroendocrine lung tumor, considered as confirmation of an ectopic origin.

### 3.6 Patient with EAS without confirmed source

#### 3.6.1 Case description patient 18

This patient was diagnosed with ACTH‐dependent hypercortisolism and had a pituitary‐MRI showing no adenoma. In 2017, this patient underwent IPSS, resulting in all ACTH ratios <2.0 pre‐CRH and <3.0 post‐CRH, consistent with an ectopic source. Basal and concurrent prolactin‐adjusted peak ACTH ratios were, respectively, 0.6 and 0.3, consistent with an ectopic source. CT imaging of the thorax and abdomen showed no evidence of an ectopic ACTH source. Medical treatment of the endogenous hypercortisolism was initiated, along with active follow‐up. A Ga‐68‐DOTA‐TOC PET/CT was performed and showed a focus in the sphenoid bone suspicious for an intra‐osseous meningioma. An ACTH‐producing meningioma is rare but is described in the literature.^22^, ^23^ Due to increasing clinical symptoms of hypercortisolism despite medical treatment and the remaining uncertainty about the source of ACTH production, a bilateral adrenalectomy was performed. It remains unclear whether, in this patient, the meningioma is the source of ectopic ACTH production or not. Repeated imaging during follow‐up, however, showed no other abnormalities suspicious for a neuroendocrine tumor, and at follow‐up imaging, the meningioma remained stable.

### 3.7 Single‐point diagnostic accuracy

When only CRH‐stimulated cases with confirmed CD were considered (*n* = 17), the single‐point diagnostic accuracy using unadjusted ACTH ratios was 82.4% (14/17), 82.4% (14/17), 88.2% (15/17), 70.6% (12/17), 76.5% (13/17), 76.5% (13/17), and 88.2% (15/17) for the *t* − 5, *t*_0_, *t* + 1½, *t* + 5, *t* + 10, *t* + 15, and *t* + 20 time points, respectively. Since two patients failed to demonstrate positive unadjusted ACTH ratios at all time points (two false‐negative patients), maximal diagnostic accuracy was achieved within the first 90 s (+1½ min) after stimulation.

### 3.8 Additional analysis: DDAVP‐stimulated IPSS procedures

Five of six DDAVP‐stimulated patients had pathology and/or remission‐confirmed CD (age 53 ± 18 years; 4 women). Two patients with unadjusted ACTH ratios <2.0 pre‐CRH and <3.0 post‐CRH were misdiagnosed with EAS. Basal prolactin‐adjusted ratios (Method 1) >1.3 and concurrent ratios (Method 2) >0.6 correctly identified all CD cases. One patient without pathology‐confirmed source had unadjusted ACTH ratios ≥2.0 pre‐CRH and ≥3.0 post‐CRH, with adjusted ratios of 3.7 (basal) and 3.0 (concurrent).

## 4 DISCUSSION

The differentiation between EAS and CD by IPSS is challenging but essential to tailor therapy. This retrospective study found that prolactin‐adjusted ACTH ratios, using both basal and concurrent prolactin concentrations and the methods described in literature,^2^, ^9^ improved diagnostic accuracy compared with unadjusted ratios. By including all consecutive patients undergoing IPSS during the study period, the risk of selection bias was minimized. Moreover, we are able to provide an extensive elaboration of the prolactin‐adjusted ACTH ratios of patients followed during a long period of time, and of the various formulas that can be used to calculate these ratios. We assessed the validity of the results and cut‐off values described in previously performed studies and assessed new methods of prolactin adjustments.

In our cohort, all patients with CD showed basal prolactin‐adjusted peak ACTH ratios >1.5, and all patients with EAS <0.6. Concurrent prolactin‐adjusted peak ACTH ratios were >1.1 in all patients with CD and <0.3 in all patients with EAS. In the literature various cut‐off values for prolactin‐adjusted peak ACTH ratios to differentiate between CD and EAS are described. The most commonly used are those of Findling et al., using basal prolactin‐adjusted peak ACTH ratios >0.8 to indicate CD and ratios <0.6 to indicate EAS; and Sharma et al., using basal prolactin‐adjusted peak ACTH ratios ≥1.3 to indicate CD and ratios ≤0.7 to indicate EAS^9^, ^10^ (Table 2). Our study results support the validity of these previously established cut‐off values. In the present study, the optimal cut‐off values for basal prolactin‐adjusted ACTH ratios (Method 1) and concurrent prolactin‐adjusted ACTH ratios (Method 2) differ, and therefore, cut‐off values cannot be used interchangeably. The prolactin‐adjusted peak ACTH ratios and the reference discriminative values should be interpreted with caution and in clinical context, given the limited size of the present cohort.

**TABLE 2:** Overview of studies and their used cut‐off values and methods to calculate the prolactin‐adjusted peak ACTH ratios.

Study	Number of patients	Confirmed by pathology and/or remission after surgery	Pituitary‐MRI performed	Stimulation method	Used calculations and cut‐off values	Results	Optimal (new) cut‐off value in study
Findling et al. (2004)^8^	47 CD^a^ 5 EAS	Yes	Yes^b^	CRH	Peak ACTH ratio of >2.0 pre‐CRH and/or >3.0 post‐CRH indicating CD.	Using peak post‐CRH ACTH ratio resulted in 44 TP (>4.8), 5 TN (<2.2), and 3 FN (1.2–2.7).	Basal prolactin‐adjusted peak ACTH ratio >0.8 indicating CD and <0.6 indicating EAS.
					Basal prolactin‐adjusted peak ACTH ratio >0.8 indicating CD and <0.6 indicating EAS.	Using basal prolactin‐adjusted peak ACTH ratios resulted in 47 TP (>0.8) and 5 TN (<0.6).	
Sharma et al. (2011)^9^	17 CD 8 EAS 4 occult^c^	Yes	Yes	CRH	Peak ACTH ratio of ≥2.0 pre‐CRH and/or ≥3.0 post‐CRH indicating CD.	Using peak post‐CRH ACTH ratio resulted in 16 TP, 7 TN, 1 FN (1.3), and 1 FP (9.5).	Basal prolactin‐adjusted peak ACTH ratio ≥1.3 indicating CD and ≤0.7 indicating EAS.
					Basal prolactin‐adjusted peak ACTH ratio >0.8 indicating CD and <0.6 indicating EAS.	Using basal prolactin‐adjusted peak ACTH ratio resulted in 17 TP (1.3–40.9), 6 TN (0.2–0.5), 1 FP^d^ (5.6), and 1 indeterminate (0.7).	
Grant et al. (2012)^13^	72 CD 10 EAS 1 indeterminate^e^	Yes	—	CRH	Peak ACTH ratio of >2.0 pre‐CRH and/or >3.0 post‐CRH indicating CD.	Using peak post‐CRH ACTH ratio resulted in 66 TP, 10 TN^f^, 6 FN, and 1 FP.	
					Basal prolactin‐adjusted peak ACTH ratio >0.8 indicating CD and <0.6 indicating EAS.	Using basal prolactin‐adjusted peak ACTH ratio resulted in 72 TP, 10 TN, and 1 FP.	
Qiao et al. (2015)^2^	38 CD 2 occult^g^	Yes	Yes	DDAVP	Peak ACTH ratio of ≥2.0 pre‐DDAVP and/or ≥3.0 post‐DDAVP indicating CD.	Using peak post‐DDAVP ACTH ratio resulted in 35 TP and 3 FN.	Concurrent prolactin‐adjusted peak ACTH ratio ≥1.2 indicating CD.
					Concurrent prolactin‐adjusted peak ACTH ratio >0.8 indicating CD and <0.6 indicating EAS.	Using concurrent prolactin‐adjusted peak ACTH ratio resulted in 38 TP.	
De Sousa et al. (2017)^14^	13 CD	In 9 out of 13	Yes	CRH	ACTH ratios of ≥2.0 pre‐CRH and ≥3.0 post‐CRH indicating CD.	Using ACTH ratios pre‐ and post‐CRH resulted in 13 TP.	
					Basal prolactin‐adjusted peak ACTH ratio >0.8 indicating CD and <0.6 indicating EAS.	Using basal prolactin‐adjusted peak ACTH ratio resulted in 13 TP.	
					Basal prolactin‐adjusted peak ACTH ratio ≥1,3 indicating CD and ≤0.7 indicating EAS.	Using basal prolactin‐adjusted peak ACTH ratio resulted in 11 TP diagnoses and 2 indeterminate results.	
					Concurrent prolactin‐adjusted peak ACTH ratio >0.8 indicating CD and <0.6 indicating EAS.	Using concurrent prolactin‐adjusted peak ACTH ratio resulted in 13 TP.	
Akbari et al. (2020)^10^	15 CD 5 EAS	Yes	Yes	DDAVP	ACTH ratio of ≥2.0 pre‐DDAVP and peak ACTH ratio ≥3.0 post‐DDAVP indicating CD.	Using ACTH ratios pre‐DDAVP resulted in 12 TP, 5 TN, and 3 FN. Using ACTH ratios post‐DDAVP resulted in 13 TP, 5 TN, and 2 FN diagnoses.	ACTH ratio of ≥1.76 pre‐DDAVP and ≥3.9 post‐DDAVP indicating CD. Concurrent prolactin‐adjusted peak ACTH ratio ≥0.33 indicating CD.
					Concurrent prolactin‐adjusted peak ACTH ratio ≥0.8 indicating CD and <0.8 indicating EAS.	Using concurrent prolactin‐adjusted peak ACTH ratio resulted in 13 TP, 4 TN, 2 FN, and 1 FP.	
Apaydin et al. (2022)^11^	49 CD 1 EAS 1 with adrenal adenomas^h^	Yes	Yes	CRH	Peak ACTH ratio of >2.0 pre‐CRH and/or >3.0 post‐CRH indicating CD.	Using ACTH ratios pre‐CRH resulted in 47 TP, 1 TN, and 2 FN. Using ACTH ratios post‐CRH resulted in 49 TP and 1 TN. The patient with adrenal adenomas had pre‐CRH ACTH ratios <2.0 and post‐CRH ACTH ratios <3.0.	Basal prolactin‐adjusted peak ACTH ratio >1.3 indicating CD and <0.7 indicating EAS.
					Basal prolactin‐adjusted peak ACTH ratio >0.8 indicating CD.	Using basal prolactin‐adjusted peak ACTH ratio resulted in 49 TP (>1.3) and 1 TN (0.7). The patient with adrenal adenomas had a basal prolactin‐adjusted peak ACTH ratio of 0.9.	
Detomas et al. (2023)^12^	25 CD 7 EAS	Yes	Yes	CRH	Peak ACTH ratio of ≥2.0 pre‐CRH and/or ≥3.0 post‐CRH indicating CD.	Using ACTH ratios post‐CRH resulted in 22 TP, 7 TN, and 3 FN.	ACTH ratio of ≥1.9 pre‐CRH and ≥2.1 post‐CRH indicating CD. Basal prolactin‐adjusted peak ACTH ratio ≥1.4 indicating CD.
					Basal prolactin‐adjusted peak ACTH ratio ≥1,3 indicating CD and ≤0.7 indicating EAS.	Using basal prolactin‐adjusted peak ACTH ratio resulted in 24 TP, 4 TN, 1 FP (1.3), and 3 indeterminate.	
Ardakani et al. (2023)^18^	8 CD 3 EAS	Yes, except one EAS^i^	Yes	DDAVP	Peak ACTH ratio of ≥2.0 pre‐DDAVP and/or ≥3.0 post‐DDAVP indicating CD.	Using ACTH ratios pre‐DDAVP resulted in 7 TP, 3 TN, and 1 FN. Using ACTH ratios post‐DDAVP resulted in 8 TP and 3 TN.	
					Concurrent prolactin‐adjusted peak ACTH ratio ≥1.3 indicating CD and ≤0.7 indicating EAS.	Using concurrent prolactin‐adjusted peak ACTH ratio resulted in 8 TP and 3 TN.	
Sprenkeler et al. (2025)	16 CD^j^ 3 EAS^k^	Yes	Yes	CRH	Peak ACTH ratio of ≥2.0 pre‐CRH and/or ≥3.0 post‐CRH indicating CD.	Using peak ACTH ratios pre‐ and post‐CRH resulted in 15 TP, 2 TN, 2 FN, and 1 FP.	Basal prolactin‐adjusted peak ACTH ratio >1.5 indicating CD and <0.6 indicating EAS. Concurrent prolactin‐adjusted peak ACTH ratio >1.1 indicating CD and <0.3 indicating EAS.
					Basal prolactin‐adjusted peak ACTH ratio >0.8 indicating CD and <0.6 indicating EAS.	Using basal prolactin‐adjusted peak ACTH ratio resulted in 17 TP (1.5–110.1) and 3 TN (0.3–0.6).	
					Basal prolactin‐adjusted peak ACTH ratio ≥1.3 indicating CD and ≤0.7 indicating EAS.	Using basal prolactin‐adjusted peak ACTH ratio resulted in 17 TP (1.5–110.1) and 3 TN (0.3–0.6).	
					Concurrent prolactin‐adjusted peak ACTH ratio >0.8 indicating CD and <0.6 indicating EAS.	Using concurrent prolactin‐adjusted peak ACTH ratio resulted in 17 TP (1.1–61.3) and 3 TN (0.1–0.3).	
					Concurrent prolactin‐adjusted peak ACTH ratio ≥1.3 indicating CD and ≤0.7 indicating EAS.	Using concurrent prolactin‐adjusted peak ACTH ratio resulted in 15 TP (1.5–61.3), 3 TN (0.1–0.3), and 2 indeterminate (1.1 and 1.2).	

*Note*: Adapted from Ref. [20].

^a^ Three of whom are index cases.

^b^ Only described for the three index cases.

^c^ Occult: IPSS results suggested EAS but the source could not be localized. These four occult cases were excluded from the analysis.

^d^ Patient with cyclic Cushing syndrome in whom the catheterization was performed when she was eucortisolemic.

^e^ This patient had only a partial biochemical cure after three transsphenoidal pituitary surgeries followed by radiotherapy treatment. A pituitary source could not be confirmed with pathology. This patient is included in the analysis.

^f^ One of whom had cyclical Cushing syndrome.

^g^ These two patients did not undergo surgical exploration; final diagnosis remained unknown. They were excluded from analysis.

^h^ This patient had a 6 mm pituitary adenoma and bilateral adrenal adenomas. Adrenal venous sampling indicated lateralization to the right adrenal gland. Right adrenalectomy was performed, after which remission was achieved.

^i^ This patient did not undergo surgery, and the diagnosis was made based on the biochemical test results. This patient is included in the analysis.

^j^ One of these patients has undergone the IPSS procedure twice, leading to 20 IPSS procedures included for the analysis.

^k^ In one of these patients, it remains unclear whether a meningioma is the source of ACTH production or not.

Table 2 summarizes previously published results on prolactin adjustment during IPSS, including the adjustment methods and cut‐off values used. A significant heterogeneity is observed among the studies, leading to difficulties in comparing study results and assessing optimal cut‐off values. Most studies confirmed the presence of an ACTH‐producing source by pathology and/or remission after surgery; however, some studies included patients in whom the ACTH‐producing source could not be confirmed according to this gold standard. Out of nine studies, three used DDAVP and the remaining six used CRH for stimulation. In five studies, prolactin‐adjusted peak ACTH ratios were calculated using basal prolactin, whereas in three other studies concurrent prolactin was used. De Sousa et al. calculated both basal and concurrent prolactin‐adjusted peak ACTH ratios. To enhance the reliability of conclusions and the generalizability of optimal cut‐off values in future analyses, standardization of study protocols is crucial.

Contrasting results are described about the potential added diagnostic value of using prolactin during IPSS. Most studies showed an improved diagnostic value when using prolactin‐adjusted peak ACTH ratios.^2^, ^9^, ^14^ Lyu et al. recently published a meta‐analysis, in which a total of 10 studies were included, showing a statistically significant improvement in sensitivity to diagnose CD when prolactin adjustments were used in IPSS compared with IPSS without prolactin adjustments.^20^ In this meta‐analysis, both calculation Methods 1 and 2 used in our study have been included in the analysis. In accordance with most of the earlier reports,^2^, ^9^, ^10^, ^12^, ^14^ our study confirmed the added diagnostic value of prolactin measurement during IPSS when basal (Method 1) and concurrent (Method 2) prolactin‐adjusted peak ACTH ratios were used. In addition to previously published studies, in our study, we examined new calculation methods including adjusting all individual ACTH ratios and then selecting the peak prolactin‐adjusted ACTH ratio (Methods 3 and 4). In contrast to adjusting only the peak ACTH ratio (Methods 1 and 2), these new strategies (Methods 3 and 4) failed to distinguish between patients with CD and patients with EAS and are therefore not recommended.

It has been suggested that corrections of ACTH ratios with prolactin concentration only have additive diagnostic value when no clear elevated ACTH ratios (<2.0 pre‐CRH and <3.0 post‐CRH) are demonstrated.^9^, ^10^ In the meta‐analysis by Lyu et al., an increased sensitivity but no statistically significant difference in specificity was demonstrated when prolactin‐adjusted peak ACTH ratios were used compared with non‐adjusted ACTH ratios.^20^ Our study showed a better diagnostic accuracy of the IPSS when prolactin‐adjusted ACTH ratios were used; we showed an increased sensitivity and specificity.

The study's main limitation is its small sample size, consistent with other studies given the rarity of Cushing syndrome and limited use of IPSS. We confirmed CD in all cases through pathology or biochemical remission and EAS in two cases through pathology. The ectopic source in one patient with EAS remains unconfirmed, a limitation we acknowledge. Peripheral prolactin levels documented before IPSS were not available for 2 patients, thereby limiting our ability to assess the presence of hyperprolactinemia in these patients. They did not show clinical features suggestive of hyperprolactinemia. One must also be cautious with the interpretation of the prolactin‐adjusted ACTH ratios of these procedures in patients with high prolactin concentrations, as they could potentially be influenced by the presence of hyperprolactinemia.

During the first IPSS of patient 13, ACTH ratios were <2.0 pre‐CRH and <3.0 post‐CRH, whereas during the second IPSS, ACTH ratios were ≥2.0 pre‐CRH and ≥3.0 post‐CRH. Given that this patient developed higher cortisol levels during the 2 years between both procedures, the observed increase in ACTH ratios is likely related to biochemical disease progression.

Differences in optimal cut‐off values between basal and concurrent prolactin‐adjusted ACTH ratios can be attributed to displacement of the catheter during the IPSS procedure and/or CRH stimulation of prolactin. In this study, stimulation of prolactin by CRH is seen. This finding is in line with other studies.^9^, ^10^, ^24^, ^25^, ^26^ Findling et al. showed a greater than 10% increase in prolactin levels in the dominant petrosal sinus in 21 out of 44 patients with CD after CRH was administered. Qiao et al. described that stimulation with DDAVP did not increase the prolactin values significantly in their study.^2^ Table 2 shows that studies using DDAVP for stimulation used concurrent prolactin‐adjusted ACTH ratios, while studies using CRH for stimulation used basal prolactin‐adjusted ACTH ratios. The same cut‐off values were used regardless of the stimulation method. More research is required to investigate the interchangeability of cut‐off values between IPSS procedures with different stimulation methods. Currently, worldwide there is a lack of CRH, leading to increased use of DDAVP. De Almeida et al. investigated the use of DDAVP as a stimulant during IPSS and concluded that it can enhance the diagnostic accuracy of the procedure.^27^

Abdallah et al. evaluated the use of fewer sampling points during IPSS, aiming to optimize both time and cost efficiency while maintaining diagnostic accuracy. Their findings suggest that sampling with as few as two time points may provide diagnostic accuracy equivalent to that of current protocols, while potentially reducing both procedure time and associated costs.^28^ De Almeida et al. demonstrated that 97.7% of the patients with a positive response following stimulation were already positive in the third minute, and all patients (100%) until the fifth minute.^29^ Our study showed that maximal diagnostic accuracy was achieved within the first 1½ minutes after stimulation. While these findings suggest that IPSS procedures with fewer sampling time points may maintain diagnostic accuracy equivalent to conventional protocols, further prospective studies are required to validate these results.

## 5 CONCLUSION

Prolactin‐adjusted ACTH ratios improved the diagnostic accuracy of IPSS compared with unadjusted ratios. Adjusting ACTH ratios with prolactin, whether basal or concurrent, is recommended to enhance differentiation between CD and EAS.

## AUTHOR CONTRIBUTIONS

**Vera E. Sprenkeler:** Conceptualization; data curation; formal analysis; investigation; methodology; resources; writing – original draft. **Antonius E. van Herwaarden:** Conceptualization; methodology; investigation; data curation; supervision; resources; writing – review and editing. **Annenienke C. van de Ven:** Methodology; writing – review and editing; data curation. **Benno Kusters:** Resources; writing – review and editing. **Sjoerd F. M. Jenniskens:** Resources; writing – review and editing. **Romana T. Netea‐Maier:** Methodology; writing – review and editing; supervision. **Joanne M. de Laat:** Conceptualization; data curation; methodology; formal analysis; investigation; project administration; resources; supervision; writing – original draft.

## FUNDING INFORMATION

The authors have nothing to disclose.

## CONFLICT OF INTEREST STATEMENT

The authors declare no conflicts of interest.

## PEER REVIEW

The peer review history for this article is available at https://www.webofscience.com/api/gateway/wos/peer-review/10.1111/jne.70066.

## CONSENT

The authors have nothing to disclose.

## Supporting information


**Data S1.** Supporting Information.

## Data Availability

The data that support the findings of this study are available from the corresponding author upon reasonable request.
