# Supplementary material for: Prolactin‐adjusted inferior petrosal sinus sampling: Pituitary and ectopic adrenocorticotropic hormone‐dependent Cushing syndrome
Source: J Neuroendocrinol. 2025 Aug 5;37(9):e70066. doi: 10.1111/jne.70066 (PMC12404901; doi:10.1111/jne.70066)
Supplement: Supplementary file 1 — Data S1. Supporting Information. [file JNE-37-e70066-s001.docx]

**Supplement 1**

**Method 1:**

Left ACTH-ratio at +1½min = ACTH-L^+1½min^/ACTH-P^+1½min^

Right ACTH-ratio at +1½min = ACTH-R^+1½min^/ACTH-P^+1½min^

Left ACTH-ratio at +5min = ACTH-L^+5min^/ACTH-P^+5min^

Right ACTH-ratio at +5min = ACTH-R^+5min^/ACTH-P^+5min^

Left ACTH-ratio at +10min = ACTH-L^+10min^/ACTH-P^+10min^

Right ACTH-ratio at +10min = ACTH-R^+10min^/ACTH-P^+10min^

Left ACTH-ratio at +15min = ACTH-L^+15min^/ACTH-P^+15min^

Right ACTH-ratio at +15min = ACTH-R^+15min^/ACTH-P^+15min^

Left ACTH-ratio at +20min = ACTH-L^+20min^/ACTH-P^+20min^

Right ACTH-ratio at +20min = ACTH-R^+20min^/ACTH-P^+20min^

The peak ACTH-ratio is the highest calculated ratio above and is used for calculating the basal prolactin adjusted peak ACTH-ratio. Prolactin of the same side (prolactin-L or prolactin-R) as the peak ACTH-ratio is used.

Basal prolactin adjusted peak ACTH-ratio = peak ACTH-ratio/(prolactin-L^t0^ or prolactin-R^t0^/Prolactin-P^t0^)

**Method 2:**

Left ACTH-ratio at -5min = ACTH-L^-5min^/ACTH-P^-5min^

Right ACTH-ratio at -5min = ACTH-R^-5min^/ ACTH-P^-5min^

Left ACTH-ratio at t0 = ACTH-L^t0^/ACTH-P^t0^

Right ACTH-ratio at t0 = ACTH-R^t0^/ACTH-P^t0^

Left ACTH-ratio at +1½min = ACTH-L^+1½min^/ACTH-P^+1½min^

Right ACTH-ratio at +1½min = ACTH-R^+1½min^/ACTH-P^+1½min^

Left ACTH-ratio at +5min = ACTH-L^+5min^/ACTH-P^+5min^

Right ACTH-ratio at +5min = ACTH-R^+5min^/ACTH-P^+5min^

Left ACTH-ratio at +10min = ACTH-L^+10min^/ACTH-P^+10min^

Right ACTH-ratio at +10min = ACTH-R^+10min^/ACTH-P^+10min^

Left ACTH-ratio at +15min = ACTH-L^+15min^/ACTH-P^+15min^

Right ACTH-ratio at +15min = ACTH-R^+15min^/ACTH-P^+15min^

Left ACTH-ratio at +20min = ACTH-L^+20min^/ACTH-P^+20min^

Right ACTH-ratio at +20min = ACTH-R^+20min^/ACTH-P^+20min^

The peak ACTH-ratio is the highest calculated ratio above and is used for calculating the concurrent prolactin adjusted peak ACTH-ratio. Prolactin of the same side (prolactin-L or prolactin-R) and time point as the peak ACTH-ratio is used.

Concurrent prolactin adjusted peak ACTH-ratio = peak ACTH-ratio/(prolactin-L^same time point^ or prolactin-R^same time point^/Prolactin-P^same time point^)

**Method 3:**

Left basal prolactin adjusted ACTH-ratio at -5min = (ACTH-L^-5min^/ACTH-P^-5min^)/(prolactin-L^t0^/Prolactin-P^t0^)

Right basal prolactin adjusted ACTH-ratio at -5min = (ACTH-R^-5min^/ACTH-P^-5min^)/(prolactin-R^t0^/Prolactin-P^t0^)

Left basal prolactin adjusted ACTH-ratio at t0 = (ACTH-L^t0^/ACTH-P^t0^)/(prolactin-L^t0^/Prolactin-P^t0^)

Right basal prolactin adjusted ACTH-ratio at t0 = (ACTH-R^t0^/ ACTH-P^t0^)/(prolactin-R^t0^/Prolactin-P^t0^)

Left basal prolactin adjusted ACTH-ratio at +1½min = (ACTH-L^+1½min^/ACTH-P^+1½min^)/(prolactin-L^t0^/Prolactin-P^t0^)

Right basal prolactin adjusted ACTH-ratio at +1½min = (ACTH-R^+1½min^/ACTH-P^+1½min^)/(prolactin-R^t0^/Prolactin-P^t0^)

Left basal prolactin adjusted ACTH-ratio at +5min = (ACTH-L^+5min^/ACTH-P^+5min^)/(prolactin-L^t0^/Prolactin-P^t0^)

Right basal prolactin adjusted ACTH-ratio at +5min = (ACTH-R^+5min^/ACTH-P^+5min^)/(prolactin-R^t0^/Prolactin-P^t0^)

Left basal prolactin adjusted ACTH-ratio at +10min = (ACTH-L^+10min^/ACTH-P^+10min^)/(prolactin-L^t0^/Prolactin-P^t0^)

Right basal prolactin adjusted ACTH-ratio at +10min = (ACTH-R^+10min^/ACTH-P^+10min^)/(prolactin-R^t0^/Prolactin-P^t0^)

Left basal prolactin adjusted ACTH-ratio at +15min = (ACTH-L^+15min^/ACTH-P^+15min^)/(prolactin-L^t0^/Prolactin-P^t0^)

Right basal prolactin adjusted ACTH-ratio at +15min = (ACTH-R^+15min^/ACTH-P^+15min^)/(prolactin-R^t0^/Prolactin-P^t0^)

Left basal prolactin adjusted ACTH-ratio at +20min = (ACTH-L^+20min^/ACTH-P^+20min^)/(prolactin-L^t0^/Prolactin-P^t0^)

Right basal prolactin adjusted ACTH-ratio at +20min = (ACTH-R^+20min^/ACTH-P^+20min^)/(prolactin-R^t0^/Prolactin-P^t0^)

The basal prolactin adjusted ACTH-ratio is the highest calculated ratio above.

**Method 4:**

Left concurrent prolactin adjusted ACTH-ratio at -5min = (ACTH-L^-5min^/ACTH-P^-5min^)/(prolactin-L^-5min^/prolactin-P^-5min^)

Right concurrent prolactin adjusted ACTH-ratio at -5min = (ACTH-R^-5min^/ACTH-P^-5min^)/(prolactin-R^-5min^/prolactin-P^-5min^)

Left concurrent prolactin adjusted ACTH-ratio at t0 = (ACTH-L^t0^/ACTH-Pt^0^)/(prolactin-L^t0^/prolactin-P^t0^)

Right concurrent prolactin adjusted ACTH-ratio at t0 = (ACTH-R^t0^/ACTH-P^t0^)/(prolactin-R^t0^/prolactin-P^t0^)

Left concurrent prolactin adjusted ACTH-ratio at +1½min = (ACTH-L^+1½min^/ACTH-P^+1½min^)/(prolactin-L^+1½min^/prolactin-P^+1½min^)

Right concurrent prolactin adjusted ACTH-ratio at +1½min = (ACTH-R^+1½min^/ACTH-P^+1½min^)/(prolactin-R^+1½min^/prolactin-P^+1½min^)

Left concurrent prolactin adjusted ACTH-ratio at +5min = (ACTH-L^+5min^/ACTH-P^+5min^)/(prolactin-L^+5min^/prolactin-P^+5min^)

Right concurrent prolactin adjusted ACTH-ratio at +5min = (ACTH-R^+5min^/ACTH-P^+5min^)/(prolactin-R^+5min^/prolactin-P^+5min^)

Left concurrent prolactin adjusted ACTH-ratio at +10min = (ACTH-L^+10min^/ACTH-P^+10min^)/(prolactin-L^+10min^/prolactin-P^+10min^)

Right concurrent prolactin adjusted ACTH-ratio at +10min = (ACTH-R^+10min^/ACTH-P^+10min^)/(prolactin-R^+10min^/prolactin-P^+10min^)

Left concurrent prolactin adjusted ACTH-ratio at +15min = (ACTH-L^+15min^/ACTH-P^+15min^)/(prolactin-L^+15min^/prolactin-P^+15min^)

Right concurrent prolactin adjusted ACTH-ratio at +15min = (ACTH-R^+15min^/ACTH-P^+15min^)/(prolactin-R^+15min^/prolactin-P^+10min^)

Left concurrent prolactin adjusted ACTH-ratio at +20min = (ACTH-L^+20min^/ACTH-P^+20min^)/(prolactin-L^+20min^/prolactin-P^+20min^)

Right concurrent prolactin adjusted ACTH-ratio at +20min = (ACTH-R^+20min^/ACTH-P^+20min^)/(prolactin-R^+20min^/prolactin-P^+10min^)

The concurrent prolactin adjusted ACTH-ratio is the highest calculated ratio above.

**Supplement 2**

*Unadjusted ACTH-ratios*

|  |  | **Confirmed source** | |
| --- | --- | --- | --- |
|  |  | Pituitary | Ectopic |
| **Unadjusted ACTH-ratios** | ≥2.0 pre-CRH and ≥3.0 post-CRH | 15 | 1 |
|  | <2.0 pre-CRH and <3.0 post-CRH | 2 | 2 |

Sensitivity = 15/(15+2) x 100% = 88.2%

Specificity = 2/(2+1) x 100% = 66.7%

Positive predictive value = 15/(15+1) = 0,94

Negative predictive value = 2/(2+2) = 0,50

*Basal prolactin adjusted peak ACTH-ratios (method 1)*

Cut-off values of Findling et al.:

|  |  | **Confirmed source** | |
| --- | --- | --- | --- |
|  |  | Pituitary | Ectopic |
| **Basal prolactin adjusted peak ACTH-ratios (method 1)** | >0.8 | 17 | 0 |
|  | 0.6-0.8 | 0 | 0 |
|  | <0.6 | 0 | 3 |

Sensitivity = 100%

Specificity = 100%

Positive predictive value = 1

Negative predictive value = 1

Cut-off values of Sharma et al.:

|  |  | **Confirmed source** | |
| --- | --- | --- | --- |
|  |  | Pituitary | Ectopic |
| **Basal prolactin adjusted peak ACTH-ratios (method 1)** | ≥1.3 | 17 | 0 |
|  | 0.7-1.3 | 0 | 0 |
|  | ≤0.7 | 0 | 3 |

Sensitivity = 100%

Specificity = 100%

Positive predictive value = 1

Negative predictive value = 1

Cut-off value from ROC curve analysis:

|  |  | **Confirmed source** | |
| --- | --- | --- | --- |
|  |  | Pituitary | Ectopic |
| **Basal prolactin adjusted peak ACTH-ratios (method 1)** | >1.0 | 17 | 0 |
|  | <1.0 | 0 | 3 |

Sensitivity = 100%

Specificity = 100%

Positive predictive value = 1

Negative predictive value = 1

*Concurrent prolactin adjusted peak ACTH-ratios (method 2)*

Cut-off values of Findling et al.:

|  |  | **Confirmed source** | |
| --- | --- | --- | --- |
|  |  | Pituitary | Ectopic |
| **Concurrent prolactin adjusted peak ACTH-ratios (method 2)** | >0.8 | 17 | 0 |
|  | 0.6-0.8 | 0 | 0 |
|  | <0.6 | 0 | 3 |

Sensitivity = 100%

Specificity = 100%

Positive predictive value = 1

Negative predictive value = 1

Cut-off values of Sharma et al.:

|  |  | **Confirmed source** | |
| --- | --- | --- | --- |
|  |  | Pituitary | Ectopic |
| **Concurrent prolactin adjusted peak ACTH-ratios (method 2)** | ≥1.3 | 15 | 0 |
|  | 0.7-1.3 | 2 | 0 |
|  | ≤0.7 | 0 | 3 |

Sensitivity = 100% *(without the indeterminate results)*

Specificity = 100%

Positive predictive value = 1 *(without the indeterminate results)*

Negative predictive value = 1

Cut-off value from ROC curve analysis:

|  |  | **Confirmed source** | |
| --- | --- | --- | --- |
|  |  | Pituitary | Ectopic |
| **Concurrent prolactin adjusted peak ACTH-ratios (method 2)** | >0.7 | 17 | 0 |
|  | <0.7 | 0 | 3 |

Sensitivity = 100%

Specificity = 100%

Positive predictive value = 1

Negative predictive value = 1

**Supplement 3**

|  | Minimum | Maximum | Mean (SD) |
| --- | --- | --- | --- |
| Basal prolactin adjusted peak ACTH-ratio, basal values at t-5min were used | 0.22 | 203.61 | 26,57 (50,12) |
| Basal prolactin adjusted peak ACTH-ratio, basal values at t0 were used | 0.31 | 110.11 | 22,70 (34,23) |

Table S1: Differences between the basal prolactin adjusted peak ACTH-ratios when t0 measurements were used as basal measurements instead of t-5min measurements.

**Supplement 4:**





Figure S1. The right sided prolactin adjusted ACTH-ratios of the IPSS procedure of patient 17, calculated with both basal (dashed line) and concurrent prolactin (continuous line), together with the right sided unadjusted ACTH-ratios (dotted line). Ratios are shown for each sampling time point.
